# Calcified Diverticula in the Setting of Per Rectal Bleed

**DOI:** 10.7759/cureus.3206

**Published:** 2018-08-27

**Authors:** Sindhura Kolli, Vahe Shahnazarian, Madhavi Reddy

**Affiliations:** 1 Internal Medicine, The Brooklyn Hospital Center, Affiliate of the Mount Sinai Hospital, Brooklyn, USA; 2 Gastroenterology and Hepatology, The Brooklyn Hospital Center, Affiliate of the Mount Sinai Hospital, New York, USA; 3 Gastroenterology and Hepatology, The Brooklyn Hospital Center, Affiliate of the Icahn School of Medicine at Mount Sinai, New York, USA

**Keywords:** calcified diverticulum, rectal bleed, gastrointestinal bleed, diverticula

## Abstract

Diverticula and calcification of tissue are both common processes; however, when occurring synergistically, they present a rare phenomenon. Our case of a 62-year-old female with frank blood per rectum revealed calcified diverticula on a non-contrast computed tomography (CT) scan. This is a diagnosis of exclusion having ruled out differentials such as calcified fecaliths, ingestion of radiopaque liquids, pills or objects, calcified infectious cysts, and neoplastic masses. After resuscitation and stabilization of the patient, the subsequent priority shifted to the assessment of the rebleeding risk. With calcification being a rare occurrence with diverticula, it is unclear how this constant irritating nidus will affect the chances of rebleeding. Thus, for this particular phenomenon, patients with calcified diverticula should be monitored with dedicated surveillance to ascertain rebleeding rates.

## Introduction

Diverticula and calcification of tissue are both common processes; however, when occurring synergistically, they present a rare phenomenon. Our case of calcified diverticula in the setting of profuse and frank per rectal bleeding was a diagnosis of exclusion having ruled out numerous other possibilities that included calcified fecaliths, ingestion of radiopaque liquids, pills or objects, calcified infectious cysts, and neoplastic masses. We recognize that this rare entity requires careful management as it can likely contribute to a high rebleeding rate.

## Case presentation

**Figure 1:**
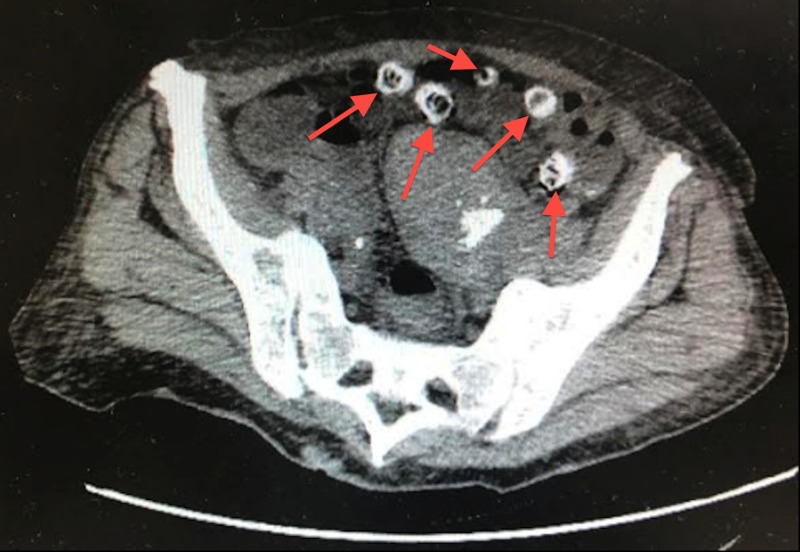
Axial computed tomography (CT) without contrast of calcified diverticula in the transverse colon causing rectal bleeding

**Figure 2:**
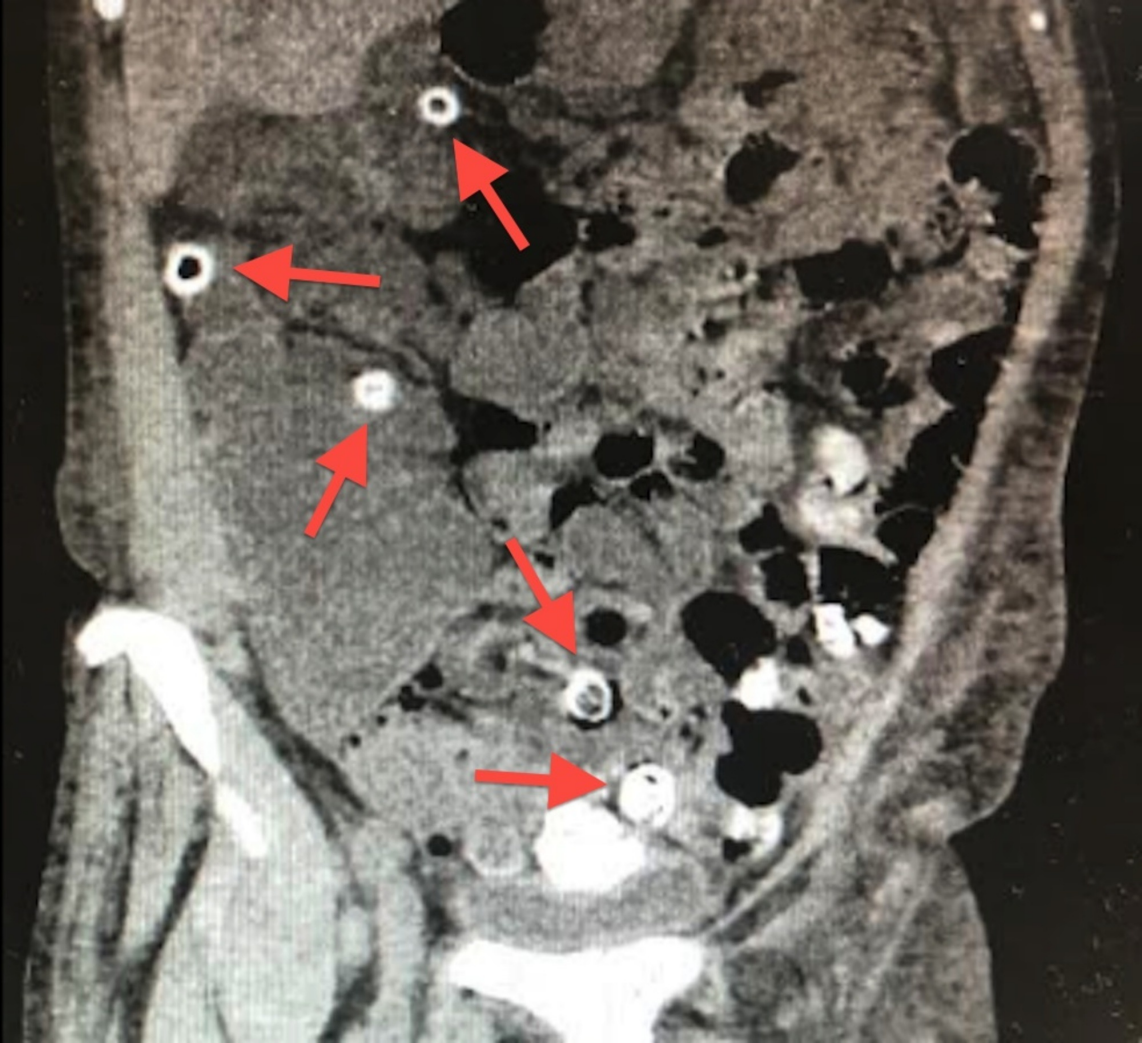
A coronal non-contrast computed tomography (CT) of calcified diverticula throughout the colon causing rectal bleeding

We present a case of a 62-year-old African American female patient admitted to the intensive care unit (ICU) with profuse rectal bleeding with a hemoglobin (Hb) of 5.3 grams per deciliter (g/dL), left lower abdominal pain, nausea, chills, and dizziness. Her extensive comorbidities include diverticulosis present for over 40 years, untreated hepatitis C, end-stage renal disease (ESRD), asthma, chronic obstructive pulmonary disease, hypertension, polycystic kidney disease, diabetes, gout, history of pulmonary embolism not on anticoagulation, cerebrovascular disease, and patent foramen ovale, while family history was significant for gastric cancer. Her medications did not include anticoagulants or non-steroidal anti-inflammatory agents (NSAIDs). She had a previous colonoscopy, two years prior in 2016, that revealed severe diverticulosis and internal hemorrhoids. She presented with a Hb of 5.3 g/dL, normal platelets and coagulation panel, low-normal corrected calcium of 8.4 milligrams/deciliter (mg/dL), and an elevated creatinine and blood urea nitrogen (BUN) due to her pre-existing ESRD. While she experienced intermittent spotting before, this episode marked the first time she experienced a profuse and rapid bleed. She was transfused two units of pure red blood cells (PRBC), started on both a proton pump inhibitor (PPI) drip and a desmopressin drip. She underwent a computed tomography (CT) of the abdomen and pelvis without intravenous (IV) contrast due to poor renal function and refusal to take per oral (PO) contrast. The CT exhibited extensive diverticula mostly in the left colon with a majority of the diverticula calcified and gastric wall thickening, best observed on the axial and coronal reconstruction below (Figures 1-2).

Calcified cysts were present in both her kidneys as well as calcified fibroids in her uterus. A previous CT without contrast, performed seven years prior, showed diverticulosis, but an absence of calcification in the above-mentioned locations at that time. She was transfused two more units of PRBC, totaling four, to eventually attain a Hb of 7.7 g/dL. The patient no longer experienced active gastrointestinal bleeding after resuscitation. Bolstered by the resolution of her symptoms in the setting of her stable Hb and vitals, she deferred an inpatient colonoscopy and endogastroduodenoscopy and agreed to follow-up as an outpatient.

## Discussion

Diverticula and calcification of tissue are both relatively common processes; however, when occurring synergistically they present a rare phenomenon. Diverticula is a phenomenon of the elderly, commonly affecting people over the age of 60 [1]. These “false” protrusions of the mucosa and submucosa layers of the colon do not include the muscularis externa layer [2] and are typically asymptomatic, unless accompanied by an infection or bleed. Diverticulitis manifests as left lower quadrant pain, fever and changes in bowel movements [1], while diverticular bleeding is subsequent to trauma of the exposed branches of the vasa recta or colonic blood vessels in a diverticulum. Generally, diverticular bleeds are chronic, intermittent and slow, bleeding at a rate less than 0.5 milliliters (ml) per minute. However, in our patient, the frank and profuse blood loss requiring four units of pure red blood cell transfusion is more consistent with a right colon diverticular bleed [2]. Calcification of these diverticula could be due to dystrophic calcification, in which there is calcification of phosphate leaking from damaged, necrotic tissue which results in radiopaqueness. This process is increased in an acidic environment despite a normal calcium level [3]. Our patient’s low-normal corrected calcium of 8.4 mg/dL, along with the presence of calcified cysts in both kidneys and calcified fibroids in her uterus points to this likely possibility. This is, however, a diagnosis of exclusion with no previous cases reported in the literature to date.

Other differentials that necessitated initial investigation included the intake of PO contrast or high-density material such as Pepto-Bismol that filled the pouches of diverticula. Blood was not suspected given the high level of attenuation seen in the concentric circles scattered throughout the colon. Ingestion of oral contrast, also known as barium sulfate, or Pepto-Bismol containing bismuth subsalicylate, commonly used to relieve heartburn, results in radiopaque images due to their high atomic numbers of 56 and 83, respectively, which readily absorbs more radiation. Another possibility was the ingestion of any of the following radiopaque products: heavy metals such as iron-containing pills, lead, mercury, medications that contain potassium [4], iodinated compounds containing 58% weighted iodine [5], antacids such as calcium carbonate, and acetazolamide. Pills with varying radiopacity are antihistamines, phenothiazines, and tricyclic antidepressants, while a previous study details that opacity is not dictated by enteric coating [4]. Ingested drug packets containing cocaine and opioids would also be visible, along with more common culprits such as ingested fish bones, toothpicks and food bolus [6]. However, our patient denied ingestion of any of the above-mentioned material including contrast and Pepto-Bismol, both during her hospital stay as well as during the five days prior to admission.

Another explanation of the CT findings could be calcified fecaliths, usually formed from a stasis of feces that have become inspissated or thick in consistency, calcified, and then difficult to dispel. They can be stuck in appendiceal openings or diverticula causing impaction and in rare cases, profuse colonic bleeding [7]. However, they usually are 1-2 in number versus the multiple ones our patient displayed. Calcified soft tissue masses or neoplastic masses with at least 30 Hounsfield units (HU) [8] will also be radiopaque, but our patient had none of the accompanying clinical features associated with a neoplastic process. Infectious calcified cysts of *Echinococcus granulosus* are usually seen in the liver and lungs and are round with smooth walls [9], while calcified larval cysts of *Taenia solium* settle in skeletal muscle, subcutaneous tissue and brain as well-circumscribed, tiny, with surrounding edema [10]. Neither were similar to our CT findings or our patient’s clinical picture leading us to conclude that the diverticula seen were calcified.

After initial resuscitation and stabilization of our patient, the next concern was the risk of rebleeding. After an initial diverticular bleed, the rate of rebleeding can up to 51% within two years of the initial bleed [11] with the main risk factor being that initial bleed. With calcification being a rare occurrence with diverticula, it should be monitored with dedicated surveillance to ascertain rebleeding rates for this particular phenomenon.

## Conclusions

After suffering from diverticula for 40 years, our patient began to develop dystrophic calcification of her abnormal protrusions that likely contributed to her profuse bleeding. The patient spontaneously stopped bleeding after symptomatic treatment that included a large volume blood transfusion, but still stands at a high risk for rebleeding due to the occurrence of her initial episode as well as the unknown recurrence rate for this rare phenomenon of calcified diverticula. Close surveillance with regular follow-up is required for patients with calcified diverticula until further cases come forward for a broader analysis.
